# Beyond the tumor: Enhancing pancreatic cancer therapy through glutamine metabolism and innovative drug delivery

**DOI:** 10.1002/ccs3.70033

**Published:** 2025-07-09

**Authors:** Min Su, Huan Qin, Jie Shen, Hao An, Yu Cao

**Affiliations:** ^1^ School of Pharmacy Qingdao University Qingdao China; ^2^ School of Basic Medicine Qingdao University Qingdao China; ^3^ Clinical Trials Center The Affiliated Hospital of Qingdao University Qingdao China

**Keywords:** DON, drug delivery systems, glutamine metabolism, pancreatic cancer, targeted therapy, tumor microenvironment

## Abstract

Pancreatic ductal adenocarcinoma (PDAC) depends a lot on how it uses glutamine to grow quickly and stay alive. Oncogenic drivers such as KRAS, c‐Myc, and HIF‐1α increase how much glutamine gets taken up and broken down. Meanwhile, the bacteria in the gut and tumor itself also affect how much glutamine is available throughout the body and near the tumor. This impacts both how the tumor grows and how the immune system can detect and respond to it. Multiple strategies have emerged to disrupt this dependence: glutamine antagonists (DON and its prodrugs DRP‐104, JHU‐083), small‐molecule glutaminase inhibitors (CB‐839), antibody–drug conjugates targeting the ASCT2 transporter, and combination regimens pairing glutamine blockade with immune checkpoint inhibitors. Nanoparticle formulations—including pH‐sensitive and PEGylated liposomes co‐delivering DON and gemcitabine—enable targeted delivery and reduce off‐target toxicity. Single‐agent treatments do not work so well because the cells can adapt. They boost enzymes such as asparagine synthetase and increase how they burn fatty acids to make up for the lack of glutamine. To overcome these escape routes, future interventions must concurrently target compensatory pathways and integrate biomarker‐driven patient selection. Combining glutamine‐targeted agents with inhibitors of asparagine synthesis or lipid oxidation, guided by multi‐omics profiling, promises a more durable therapeutic benefit and lays the groundwork for personalized treatment of PDAC.

## Graphical abstract

The top row shows key drivers‐KRAS, c‐Myc and HIF‐1a‐alongside gut and tumor microbiota, all converging on glutamine metabolism in PDAC. At the center, glutamine handling is depicted as the linchpin of tumor growth, biosynthesis and immune modulation. The green panel lists major intervention strategies: DON‐loaded nanoparticles, prodrugs (DRP‐104/JHU‐083), the glutaminase inhibitor CB‐839, and ASCT2‐targeted ADCs plus immunotherapy combinations. Finally, the two yellow‐orange ovals at the bottom highlight adaptive escape routes—upregulated asparagine synthesis and enhanced fatty‐acid oxidation‐that blunt single‐agent glutamine inhibitors and underscore the need for co‐targeting compensatory pathways.

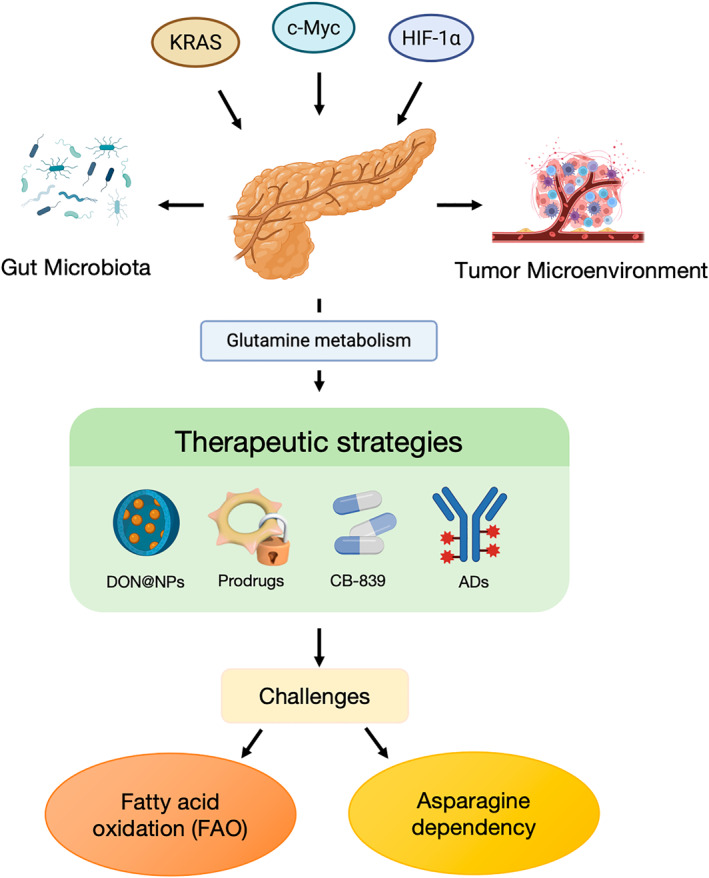

## 1 INTRODUCTION

Known as the “king of cancer,” pancreatic cancer (PC) is one of the common malignant tumors of the digestive tract, and its incidence and mortality are rising rapidly, with cases increasingly occurring in younger individuals.^1^ PC ranks among the five leading causes of cancer‐related mortality, according to data from the 2023 oncology journal Clinicians Journal of Cancer.^2^ According to the National Cancer Center 2024 National Cancer Report of China, new cases of PC in China still rank the 10th among malignant tumors in China.^3^ At present, the treatment of PC mainly relies on surgical treatment, radiotherapy, chemotherapy, and other traditional treatments; however, outcomes remain dismal, with a 5‐year overall survival rate of approximately 13%,^4^, ^5^ and, following radical resection for PC, a rate of only 8%.^6^

Pancreatic ductal adenocarcinoma (PDAC) is the most common malignant type of PC and the most aggressive and malignant one, accounting for more than 90% of all PC cases.^7^ With a poor prognosis, it remains one of the most difficult challenges in oncology.^8^ Currently, the most effective treatment method for PDAC patients is radical surgical resection.^9^ For patients who are ineligible for surgery or who have undergone resection, adjuvant treatment relies on multi‐agent chemotherapy—most commonly modified FOLFIRINOX or gemcitabine/nab‐paclitaxel—often combined with radiotherapy or chemoradiation to improve local control.^10^. Even so, median overall survival remains only 22–28 months, and treatment‐related toxicities frequently force dose reductions.^11^ Targeted agents and immunotherapies have produced only marginal gains.^12^ These issues show that we really need other options, like treatments that focus on how PDAC cells use glutamine. This could help get past their natural resistance to chemo and their heavy energy needs.^13^

The NCCN Clinical Practice Guidelines for PC (2024. V1 edition) emphasize resectable PC and postoperative adjuvant therapy and locally advanced PC and metastatic PC include gemcitabine, fluorouracil, oxaliplatin, paclitaxel, and irinotecan. The most commonly used are nab‐paclitaxel/gemcitabine regimen and FOLFIRINOX.^14^ Based on the NAPOLI 3 study,^15^ NALIRIFOX is also used in the first‐line treatment of PC, the study compared NALIRIFOX regimen and nab‐paclitaxel/gemcitabine regimen, and the results show that NALIRIFOX improved median survival (up to 11.1 months); this study provides a new possibility to further improve the treatment of PC. Despite the continuous innovations in chemotherapy regimens, the overall survival time of PDAC patients is still not more than 1 year, and drug resistance is the main reason.^16^ Furthermore, studies have shown a strong link between epithelial–mesenchymal transition (EMT) and resistance to gemcitabine in PDAC.^17^ Therefore, it is important to find the key mechanism of chemotherapeutic resistance in PC to overcome drug resistance and prediction of chemotherapy effect.

PC is special in its stromal components, which are more prominent than other solid tumors. In PC, it has been noticed that there is a big reaction in the tissue before fibrosis really kicks in. This causes a lot of dense fibrous tissue to form around the tumor cells, creating a pretty unique kind of fibrosis in the tumor's surrounding environment, known as the TME.^18^, ^19^ Prostate cancer cells often become resistant to chemotherapy, in part because the surrounding tumor microenvironment supports their survival.^20^, ^21^ Gln metabolism in the tumor environment is really active. It helps fuel the cancer cells and creates a supportive setting for them while also making it harder for chemotherapy to work effectively.^22^ Other parts of the tumor microenvironment, such as cancer‐associated fibroblasts (CAF)^23^ and immune cell,^17^ also influence how well chemotherapy works and can contribute to resistance.

This review looks at how glutamine (Gln) is involved in PC and updates on recent treatments that target Gln. It covers both small‐molecule drugs and antibody‐based strategies. We explore the key features of Gln metabolism in PC and analyze advances in therapies aimed at Gln, including inhibitors and antibodies. By understanding the role of Gln in PC, we hope to identify new treatment options and strategies. These insights could lead to more effective drugs, help overcome resistance, and improve how we approach treatment for PC.

## 2 THE ROLE OF GLUTAMINE METABOLISM IN TUMORS, ESPECIALLY IN PC

### 2.1 Understanding Gln metabolism in different tumors

Gln is the most common amino acid found in our blood and muscles. It is really important for many types of cancers—such as pancreatic, lung, liver, ovarian, and breast cancers—because these tumors rely heavily on Gln to survive and keep growing. Proliferating cancer cells rely on Gln as the main source of energy to meet the tricarboxylic acid (TCA) cycle.^24^ Studies have demonstrated that tumors depend on Gln transporter proteins to facilitate Gln uptake from the extracellular environment. Once internalized, Gln is converted into glutamate (Glu) by glutamate dehydrogenase (GLUD1), which then drives the production of α‐ketoglutaric acid (α‐KG) to fuel the TCA cycle and maintain cellular energetics.^25^

However, the reliance on Gln metabolism varies significantly across cancer types, driven by genetic and phenotypic differences.^26^ For example, in breast cancer, the dependency on Gln differs by subtype, with triple‐negative breast cancer (TNBC) displaying a particularly strong dependence. Recent research has shown that the increased expression of the glutamine (Gln) transporter SNAT2/SLC38A2 in TNBC bolsters the cells' ability to uptake Gln and withstand oxidative stress. This adaptation is linked to more aggressive tumor growth and poorer patient outcomes.^27^ Beyond just fueling proliferation, glutamine plays a role in driving breast cancer progression by influencing various metabolic pathways and strengthening antioxidant defenses.^28^

In PC, tumor cells demonstrate a particularly high dependency on Gln metabolism, which not only supports the tricarboxylic acid (TCA) cycle but also supplies essential building blocks for swift cell division.^22^ This intense reliance reveals underlying metabolic irregularities in PC cells, such as abnormal activation of glycolytic and glutaminolytic pathways that together promote tumor growth and metastasis.^29^, ^30^ What's more, glutamine metabolism within the tumor microenvironment (TME) aids in the survival of CAF (CAFs) and influences immune cell infiltration, further contributing to therapy resistance. Importantly, studies indicate that depleting Gln in PC cells can trigger ferroptosis, pointing to its potential as a targeted therapeutic approach.^31^

In ovarian cancer, glutamine metabolism is closely linked to tumor aggressiveness and poor prognosis. It impacts key processes including cell cycle regulation, apoptosis, and overall metabolic flux while also activating signaling pathways such as mTOR/S6 and MAPK that drive tumor growth and spread.^32^, ^33^ These pathways not only sustain tumor proliferation but also help tumors resist chemotherapy by maintaining redox balance.

Colorectal cancer (CRC) cells generally also exhibit a dependence on Gln metabolism, particularly in KRAS‐mutant subtypes. KRAS mutations increase Gln uptake and utilization, supporting tumor cell proliferation and survival.^22^, ^34^, ^35^, ^36^ Oncogenic KRAS also reroutes Gln through a non‐canonical pathway involving GOT1 and MDH1 to sustain redox balance and fuel tumor growth.^22^ In KRAS‐driven PC models, simultaneous inhibition of ERK signaling and autophagy markedly improved antitumor efficacy.^37^ Building on these findings, next‐generation KRAS G12C inhibitors (e.g., sotorasib) are being combined with metabolic agents such as glutaminase inhibitors to jointly target the Gln addiction driven by KRAS mutations.^38^ Moreover, increased Gln dependency, coupled with the already high demand for Gln in normal intestinal cells, can create localized nutrient depletion, leading to intratumoral Gln starvation and metabolic stress. Furthermore, Gln catabolism facilitates the biosynthesis of α‐KG, which complements TCA cycle intermediates and sustains cellular energetics.^39^, ^40^ Recent stable‐isotope tracing and flux‐analysis studies have demonstrated that PDAC cells exhibit significantly higher Gln uptake rates and increased glutaminolysis flux compared with other solid tumors, a phenomenon driven by upregulation of SLC1A5 and GLS1, and underscoring the exceptional Gln dependence of PC.^41^

In gastric cancer, Gln metabolism is significantly upregulated in tumor tissues, supplying energy and biosynthetic precursors to cancer cells.^42^ Similarly, liver cancer has been shown to depend on Gln metabolism, with studies highlighting the regulatory role of liver‐specific microRNA miR‐122 in Gln metabolic pathways.^43^

In lung cancer, genes involved in Gln metabolism, such as EPHB2, are overexpressed and play a central role in promoting tumor growth. Although the precise mechanisms remain unclear, EPHB2 likely modulates the immune response via the Gln metabolic pathway.^44^ Studies have shown that modulation of Gln metabolism, combined with an approach to increase SIRT4 protein expression, can significantly suppress tumor growth in treating mice of lung cancer H1299 cell transplantation model.^45^ Additionally, the Gln transporter SLC1A5 has been identified as a diagnostic biomarker in lung cancer, mediating Gln uptake and serving as a therapeutic target. This research on SLC1A5 has inspired efforts to identify similar biomarkers for PC treatment.^46^

**FIGURE 1:**
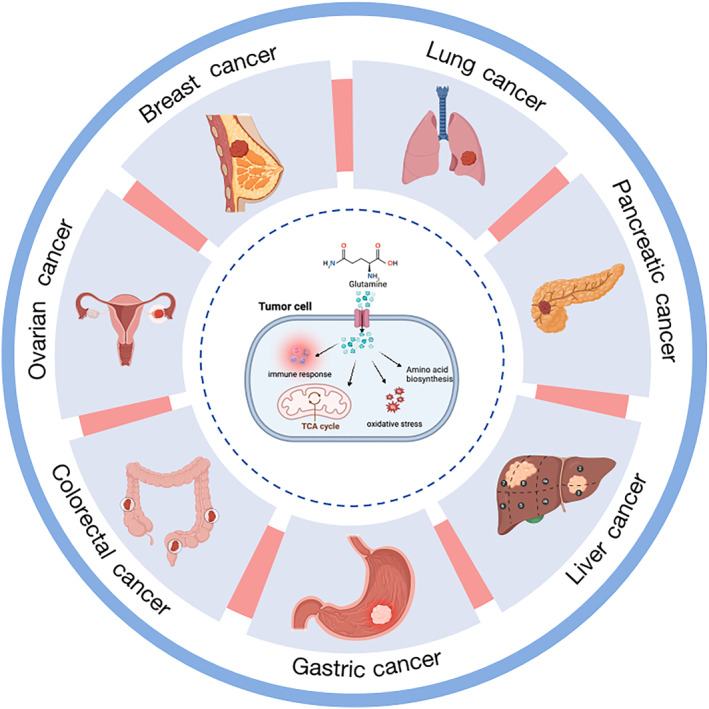
The role of Gln metabolism across different tumor types. This diagram highlights the critical involvement of Gln metabolism in multiple cancers, including pancreatic, lung, breast, ovarian, colorectal, gastric, and liver cancers. Within tumor cells, Gln is metabolized through the TCA cycle, supporting biosynthesis, energy production, and maintaining redox homeostasis. This metabolic dependency underscores Gln as a key therapeutic target, with potential strategies focusing on disrupting Gln utilization to inhibit tumor growth and progression.

In conclusion, Gln metabolism plays diverse and multifaceted roles across cancer types, fueling tumor growth, sustaining metastatic potential, and driving resistance to therapy. As shown in Figure 1, Gln metabolism across various tumor types underscores its influence on these critical processes. Its critical involvement in maintaining cellular energetics and biosynthesis underscores its potential as a therapeutic target. Future research should focus on uncovering biomarkers of Gln dependency, exploring synergistic therapies that combine Gln inhibitors with other treatments, and addressing challenges in translating preclinical findings into clinical practice.

### 2.2 Unique mechanism of action of Gln metabolism in PC

Unlike other cancers, PC cells rely on a distinct mechanism to promote the TCA cycle. Gln‐derived aspartate (Asp) is transported into the cytosol, where it is converted to oxaloacetate (OAA) by aspartate transaminase (AST), OAA is subsequently converted into malate and then pyruvate. Malate plays an essential role in generating NADPH, which helps increase the NADPH/NADP^+^ ratio and supports the cell's redox balance.^22^ This dependence on glutamine metabolism emphasizes its importance in helping PC cells survive and grow, making it a key area of focus in current research. By exploring the unique pathways of glutamine metabolism in PC, scientists hope to identify new biomarkers and gain a better understanding of how tumors grow and progress, eventually paving the way for innovative treatment options.

The development of PC is closely tied to how glutamine metabolism is regulated. Enzymes such as GLUD1 and glutamine synthetase (GS) are important in controlling how glutamine is produced and broken down. For example, GLUD1 helps break down glutamine, influencing how much energy PC cells can generate, whereas GS determines how much glutamine the cells need, which affects their growth and division. Studies have shown that these enzymes are essential in supporting tumor growth and can be potential targets for therapy.^47^, ^48^

In addition, molecular mechanisms also play a major role in the progression of PC. The mTOR signaling pathway, a major regulator of cellular metabolism, is particularly important for PC cell proliferation.^17^, ^49^ Transport proteins such as L‐type amino acid transporter 2 (LAT2) influence the mTOR pathway that depends on glutamine, modulating how glutamine metabolism functions within the cells. This regulation can make PC cells less sensitive to gemcitabine, a common chemotherapy drug while also promoting increased growth and invasive behavior in the tumors.^50^

Furthermore, PC cells, the transcription factor c‐Myc is found to be overactive, which helps kickstart the glutamine (Gln) metabolic pathway. When c‐Myc levels are high, they boost the growth, invasion, spread, blood vessel formation, and the ability of cancer cells to hide from immune responses. On the other hand, lowering c‐Myc activity can make cancer cells more vulnerable to chemotherapy and improve how well immunotherapy works. This emphasizes just how important c‐Myc is in the progression of PC.^51^ Specifically, c‐Myc directly controls how SLC1A5 manages Gln transport, causing changes in the cell's metabolism, which links the cancer's growth with its dependence on Gln.^25^, ^52^ These changes affect how quickly cancer grows, how likely it is to invade nearby tissues, and how resistant it is to drugs. By targeting the glutamine metabolic pathway, we could slow down cancer growth, reduce its spread, and help make treatments more effective. Understanding the key enzymes and pathways involved in Gln metabolism is not only essential to understanding how PC develops but also provides a promising foundation for creating new therapies.

## 3 Gln METABOLISM IN THE PDAC MICROENVIRONMENT: ROLES, THERAPEUTIC OPPORTUNITIES, AND RESISTANCE MECHANISMS

### 3.1 The role of Gln metabolism in the microenvironment of PC

PC has a very unique microenvironment called the tumor microenvironment (TME). This includes a thick network of extracellular material, cells called CAF (CAFs), and various immune cells that constantly communicate with tumor cells, including a special type called cancer stem cells (CSCs).^53^ This TME plays a big role in why PC often resists treatment. There are several reasons behind this resistance.

#### 3.1.1 Fibrotic barrier and hypoxia

The fibrotic tissue in PC makes it harder for chemotherapy drugs to reach the tumor cells. This dense, fibrous barrier is created by CAF (CAFs) and leads to low oxygen levels inside the tumor environment,^54^ which can make treatments less effective.^55^, ^56^ To improve drug delivery, scientists are exploring ways to target or break down this fibrous matrix. For example, using enzymes that degrade hyaluronic acid or collagen fibers can help chemotherapy drugs penetrate better, potentially making treatments more successful.^57^, ^58^

#### 3.1.2 CAF‐induced immune suppression

Pancreatic stellate cells (PSCs) have the ability to turn into CAF (CAFs). These CAFs release proteins that build up a dense, fibrous environment around the tumor, creating a low‐oxygen, or hypoxic, setting that makes chemotherapy less effective. This kind of environment also blocks immune cells from getting in and helps the tumor evade the immune system, making treatment even harder.^59^, ^60^ Recent research suggests that if we can stop PSCs from becoming CAFs or prevent CAFs from producing these ECM proteins, we might improve how well chemotherapy works.^61^, ^62^ Moreover, new treatments that target the tumor's supporting tissue, such as small molecules or antibody‐based therapies, seem promising for breaking down this stroma and boosting the success of existing treatments.^63^, ^64^

#### 3.1.3 Metabolic adaptation under hypoxia

In PC, the tumor environment is a busy place where cancer cells compete with surrounding stromal and immune cells for oxygen and nutrients. To survive, these cancer cells adapt their metabolism, especially in how they process glucose, lactate, and glutamine. These changes help them stay stable and even resist chemotherapy. When oxygen levels drop, cancer cells often boost glycolysis and glutaminolysis—ways of breaking down nutrients to keep going—even if nutrients are scarce, which is part of what makes them so difficult to eliminate.^65^, ^66^, ^67^ Researchers are exploring treatments that block these metabolic adaptations, such as drugs that inhibit lactate dehydrogenase or interfere with glutamine metabolism, which could make cancer cells more vulnerable to chemotherapy. In addition, reducing hypoxia—or low oxygen—in the tumor environment might help drugs work better and boost the immune system's ability to fight the cancer.

#### 3.1.4 Immunosuppressive effects of gemcitabine

Immunosuppression and the infiltration of inflammatory cells in PC, along with the immune‐suppressing effects of gemcitabine, can make it harder for chemotherapy to work. Although this suppression might help reduce some side effects, it also weakens the body's ability to fight the tumor, which can lead to resistance against treatment.^68^ To address this, scientists are exploring combination therapies that include immune checkpoint inhibitors, such as PD‐1 or PD‐L1 blockers, to boost the immune system and make gemcitabine more effective.^69^, ^70^ Besides, combining chemotherapy with agents that stimulate the immune system could help change the tumor microenvironment from one that suppresses immune activity to one that encourages it, potentially leading to better treatment results.^71^

#### 3.1.5 Metabolic crosstalk between CAFs, inflammatory cells and tumor cells

CAFs in PDAC engage in metabolic crosstalk with both tumor and inflammatory cells, secreting alanine via autophagy‐driven proteolysis that is taken up by cancer cells and converted into TCA intermediates to sustain bioenergetics under nutrient stress.^72^ At the same time, stromal macrophages release IL‐6 and other cytokines that upregulate enzymes involved in alanine and Gln metabolism in both CAFs and tumor cells, amplifying this nutrient support.^73^ Blocking CAF autophagy or inhibiting alanine transport disrupts this feed‐forward loop, restores gemcitabine sensitivity, and enhances the efficacy of Gln‐targeted agents in preclinical PDAC models.^72^ Moreover, this metabolic exchange drives desmoplasia: CAF‐derived alanine and cytokine signals promote collagen and fibronectin deposition, reinforcing the fibrotic stroma and further limiting drug penetration.^74^

These findings highlight the complex interactions within the TME of PC, underscoring the need for therapeutic strategies that not only target tumor cells but also modify the supportive stromal components, including ECM, CAFs, and immune cells, to overcome the challenges of drug resistance. Combining conventional chemotherapy with strategies that target the TME—such as metabolic reprogramming, ECM degradation, or immune modulation—could significantly enhance treatment outcomes for PDAC.

Gln plays a critical role within the TME, functioning as an essential metabolic substrate for PC cells by supporting intracellular biosynthesis and energy production. It is worth noting that under the influence of PC cells, CAFs are induced to synthesize and release Gln, which is then consumed by cancer cells to promote proliferation and drug resistance.^75^ Gln not only affects tumor cell metabolism but also modulates the function of PSCs and the Wnt signaling pathway.^76^, ^77^ Although recent research has identified the Gln metabolic pathway as a potential therapeutic target for PC,^78^ here is still limited focus on how Gln metabolism interacts with other pathways, such as hypoxia‐inducible factor (HIF) signaling, which could exacerbate drug resistance mechanisms. Emerging studies also suggest that CAF‐induced ECM production and hypoxia create not only physical barriers but also activate gene pathways that enhance tumor resilience. For instance, the hypoxic TME may activate HIF signaling pathways, which in turn provide a survival advantage under chemotherapy. It is worth noting that studies reported that hypoxia‐driven stabilization of HIF‐1α enhances Gln metabolism by upregulating glutaminase expression and Gln transporter activity, thereby supporting redox homeostasis and cell survival under low‐oxygen stress.^79^, ^80^ Additionally, CAFs contribute to immunosuppression by secreting immunomodulatory factors, which warrants further research to determine if targeting immune checkpoints within the TME could reverse this resistance. In summary, although targeting Gln metabolism is a promising therapeutic approach, a more comprehensive strategy that addresses interconnected resistance mechanisms—such as ECM remodeling, HIF signaling, and CAF‐mediated immunosuppression—is essential to improve drug efficacy and overcome resistance in PC.

### 3.2 Gut microbiota and systemic regulation of Gln

Gut microbiota crosstalk regulating Gln levels and therapeutic response: Gut bacteria make or use Gln, changing its levels in the blood and inside tumors. In PDAC mice, giving probiotics brought Gln back up in both blood and tumor and made gemcitabine work better.^81^, ^82^ In patient samples, higher amounts of the bacterial metabolite indole‐3‐acetic acid go along with better responses to chemotherapy and immunotherapy, suggesting that it changes how tumors handle Gln.^83^ These findings point to using probiotics or fecal transplants to improve Gln‐targeted treatments in PC.

### 3.3 Inhibition of Gln and its pathway may enhance the sensitivity of PC to chemotherapeutic drugs

Gln is the most abundant circulating amino acid in blood and muscle and has a high uptake in different types of tumor cells. Human cells generally tend to feed through oxidative phosphorylation pathway, whereas tumor cells tend to feed metabolism such as glycolysis.^84^ To better illustrate this metabolic shift, a diagram depicting the contrasting metabolic pathways—oxidative phosphorylation in normal cells versus glycolysis in tumor cells—would aid reader comprehension.

**FIGURE 2:**
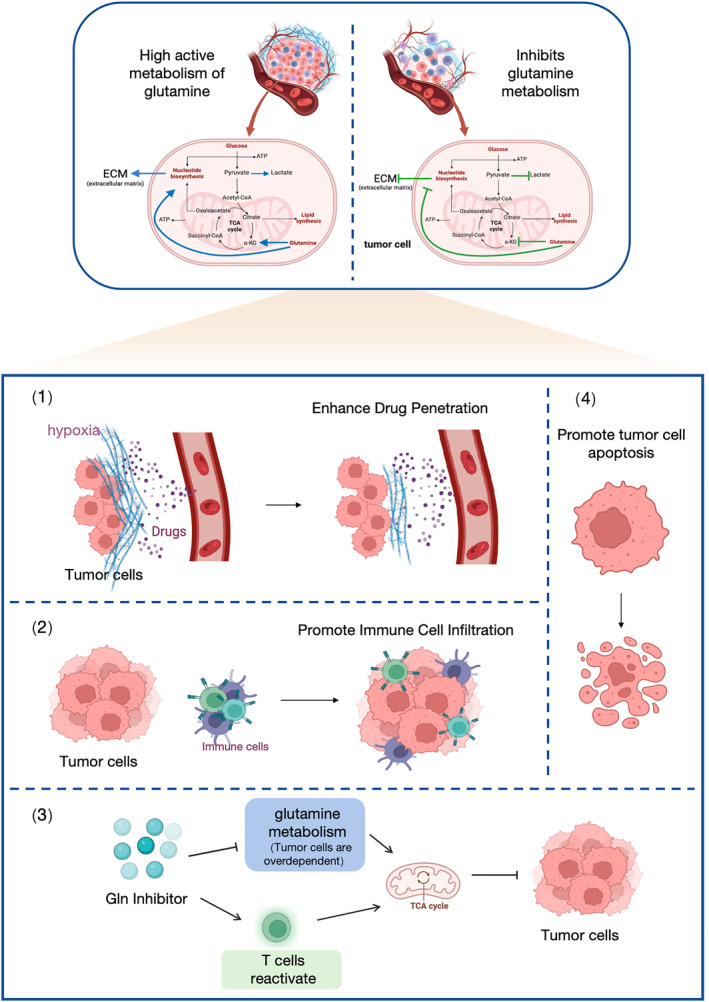
Mechanisms and therapeutic effects of Gln metabolism modulation in pancreatic cancer. The top panel illustrates the highly active Gln metabolism in tumor cells and the corresponding effects of inhibiting this metabolic pathway. Tumor cells depend on Gln for biosynthesis, energy production, and maintaining redox balance, which supports tumor growth and survival. Gln metabolism inhibition disrupts these processes, reducing tumor cell viability. The lower panel highlights therapeutic outcomes: (1) enhancing drug penetration by targeting the extracellular matrix (ECM) and overcoming hypoxia; (2) promoting immune cell infiltration into the tumor microenvironment (TME); (3) reactivating T cells through Gln inhibition, which restores antitumor immune responses; and (4) inducing tumor cell apoptosis. These strategies collectively demonstrate the potential of targeting Gln metabolism to improve PC therapies.

Metabolic rearrangements in tumors allow Gln to drive the complement pathway, promoting the TCA cycle and maintaining intracellular redox balance while providing carbon and nitrogen for biosynthetic macromolecules which required for cell division.^85^ In PC, tumor cells are more dependent on Gln metabolism to support tumor growth, exhibiting hypoxia, nutrient depletion, and oxidative stress.^86^ Therefore, inhibiting Gln metabolism may improve the efficacy of antitumor drugs and prevent drug resistance caused by metabolic remodeling. Using Gln antagonists can inhibit Gln metabolism, which inhibit energy supply in cancer cells by covalently and irreversible binding to Gln metabolic enzymes, and then inhibit the growth of cancer cells.^87^ Moreover, Gln antagonists can also reshape activated immune cells through metabolic pathways. Unlike the anaerobic glucose metabolism required to inhibit cancer cell growth, such antagonists can preferentially upregulate mitochondrial oxidative phosphorylation in effector T cells, reactivating low‐activated T cells in cancer tissues due to insufficient energy supply, to fuel the TCA cycle and maintain the redox state.^88^, ^89^ Although, the effect of Gln antagonists on the TME of PC has been studied, its research in reversing the sensitivity of resistance to chemotherapy drugs in PC patients has not been reported, and more thorough exploration is still needed (Figure 2).

Although Gln antagonists can both starve PC cells and reinvigorate tumor‐infiltrating T‐cells, their efficacy as single agents is often blunted by tumor metabolic plasticity. In response to Gln deprivation, tumor cells boost asparagine synthesis by upregulating asparagine synthetase (ASNS), allowing aspartate to be converted into asparagine to replenish TCA cycle intermediates and support nucleotide and protein biosynthesis despite Gln limitation.^90^ At the same time, fatty acid oxidation (FAO) is ramped up through increased expression of carnitine palmitoyltransferase 1A (CPT1A) and other β‐oxidation enzymes, which break down lipids to generate ATP and NADPH when Gln is scarce.^91^ These compensatory pathways act in concert to blunt the efficacy of single‐agent Gln inhibitors, highlighting the need for combination strategies that simultaneously target ASNS and FAO to overcome metabolic plasticity.

## 4 A TARGETED THERAPEUTIC STRATEGY BASED ON THE Gln METABOLIC PATHWAY

### 4.1 Gln antagonist DON and its prodrugs

Inhibition of Gln metabolism may enhance antitumor drug efficacy and prevent drug resistance due to metabolic remodeling. Gln inhibitors covalently and unstable bind Gln metabolic enzymes to inhibit energy supply in cancer cells, and then inhibit cancer cell growth.^87^ Moreover, Gln inhibitors can also reshape activated immune cells through metabolic pathways. Unlike anaerobic glucose metabolism, which is required to inhibit cancer cell growth, such inhibitors can preferentially upregulate mitochondrial oxidative phosphorylation in effector T cells, reactivate low activated T cells in cancer tissues due to insufficient energy supply, fuel the TCA cycle, and maintain the redox state.^89^, ^92^

#### 4.1.1 Application of DON and its prodrugs in tumor therapy

DON (6‐diazo‐5‐oxo‐l‐norleucine), a Gln antagonist developed for the treatment of solid tumors, was originally isolated from the fermentation broth of *Streptomyces* in the 1950s, which competitively binds to the active site of glutamate in vivo and then forms a covalent adduct that irreversibly inhibit the enzymatic active.^93^, ^94^ The diazadecone group of DON is stable under physiological conditions and triggers the release of nitrogen only when protonated under certain conditions.^95^ At low concentrations, DON can inhibit Gln‐utilizing enzyme and a variety of transglutaminases involved in de novo synthesis of purine and pyrimidine,^96^, ^97^, ^98^ coenzyme synthesis,^99^ amino acid synthesis,^100^ and hexosamine production.^101^ At high concentrations, DON simultaneously acts as a substrate for several amino acid transporters, transaminases and inhibitor.^98^ DON plays an important role in tumor cells, and due to its role as a Gln antagonist, it can influence the metabolic pathways of tumor cells and thus their growth and survival. This makes DON a potential drug research object for the treatment of tumors.

When given a high‐dose intermittent dosing regimen, DON may lead to adverse effects in organs such as the gastrointestinal tract, bone marrow, heart, kidney, and liver.^102^ However, by modifying the DON formation prodrug, gastrointestinal exposure decreases, thus reducing gastrointestinal toxicity. These prodrugs remain intact and inactive when circulating in plasma, that is, add profragments preferentially cut by enzymes enriched in the tumor (such as histone deacetylase (HDAC) or cathepsin), and then selectively release DON in the target tissue, thereby reducing the dose and increasing the therapeutic index.^95^, ^103^ JHU‐083, a prodrug of DON, is an orally effective, selective glutaminase antagonist consisting of amine and carboxyl groups of a leucine amide and an ethyl ester DON.^104^ JHU‐083 was found to be highly active in a mouse model in vivo and has important therapeutic effects on the immune system.^105^ Furthermore, blockade of Gln metabolism by JHU‐083 induces a transition of CD8 + tumor‐infiltrating lymphocytes to a more active long‐lived phenotype.^106^ JHU‐083 is significantly effective in targeting Gln metabolism in preclinical models of glioma and measurably reduces mTOR signaling.^107^ Moreover, combining JHU‐083 with anti‐PD‐1 antibodies reshapes the tumor microenvironment—reducing suppressive myeloid populations and enhancing CD8^+^ T‐cell infiltration—thereby improving checkpoint‐blockade efficacy.^87^ To date, no studies have investigated the use of this drug for the treatment of PC. DRP‐104 affects multiple metabolic pathways in tumors, including reduced Gln flux into the TCA cycle.^108^ It starved cancer cells by mimicking Gln, broadly inhibiting all the metabolic pathway using Gln.^109^ Previous studies have shown that DRP‐104 treatment alone reduces the growth rate of PDAC in a mouse model of PC. The study found that this process did not cause changes in the immune system, and the number of infiltrating T cells was very low regardless of DRP‐104 treatment, and DRP‐104 works independently of the immune system.^86^ As DRP‐104 is now for clinical trials, and researchers have great expectations for its mechanism of action to precisely target therapeutic targets, an effort to expand the treatment time window and bring hope for more patients.

#### 4.1.2 Exploring the association between DON and PC based on biological Information

**FIGURE 3:**
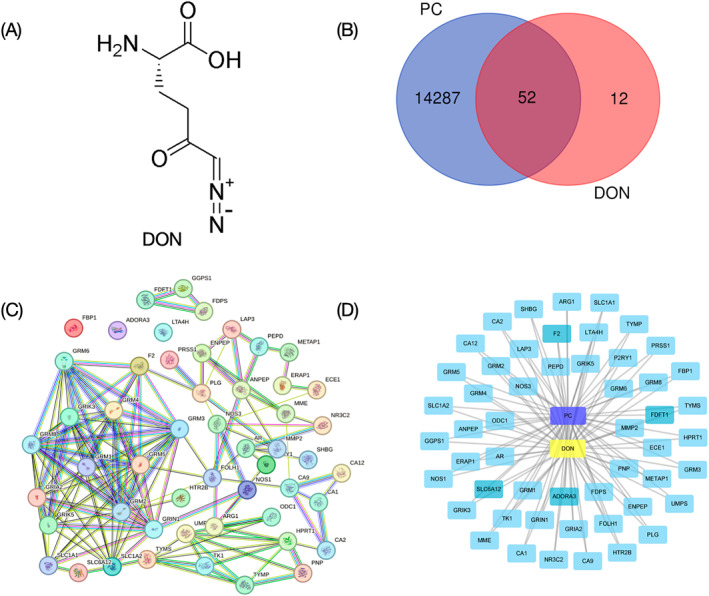
Exploring potential DON targets for pancreatic cancer treatment. (A) The chemical structure of DON. (B) A Venn diagram representing the intersection target of DON and pancreatic cancer. (C) This part maps out the protein–protein interaction network of intersected targets, where points symbolize proteins and edges indicate protein associations. (D) The complex interactions among the intersected targets of DON and PC. In the diagram, PC is represented by purple nodes, DON by yellow nodes, with blue rectangle nodes marking targets. Edges signify interactions between DON, PC, and cross‐point targets.

DON is a Gln antagonist, which can irreversibly inhibit the decomposition of Gln DON has good anticancer activity (especially in PC), and can reduce the self‐renewal potential and metastasis ability of tumor cells. The chemical structure of DON is shown in Figure 3A.

##### 4.1.2.1 Potential target screening

The Swiss Target Prediction online tool (http://www.swisstargetprediction.ch/) was utilized to identify the target genes of DON. A total of 64 related targets were identified.

To procure targets for PC, we employed the GeneCards database (https://www.genecards.org/, *n* = 13,778), OMIM database (https://omim.org/, *n* = 503), and NCBI (https://www.ncbi.nlm.nih.gov/gene, *n* = 2956). Following duplicate removal, we acquired 14,364 related targets.

The Venny 2.1 online tool (https://bioinfogp.cnb.csic.es/tools/venny/) was then used to intersect the targets of PC with the target genes of DON, yielding 52 overlapping targets as illustrated in Figure 3B,D. For target normalization, the UniProt database (https://www.UniProt.org/) was utilized, searching with the gene s within the “Human” species category.

##### 4.1.2.2 Building PPI networks

The STRING online tool (https://cn.string‐db.org/) was used to perform a protein–protein interaction (PPI) analysis on the intersecting targets, using a species filter of “Homo sapiens” and a minimum interaction score of 0.400. In the generated network, genes and their interactions were depicted as nodes and lines, respectively. After excluding the five target proteins that lacked interactions, resulting in a PPI network composed of 52 nodes (representing active proteins) and 146 edges (indicating interactions between these active proteins and other proteins) (Figure 3C). With an average node degree of 5.62 and an average local clustering coefficient of 0.542, the top 10 targets were selected for enrichment mapping based on the quantity of target interactions and binding capacity. The s and annotations of these 10 targets are shown in Table 1.

**TABLE 1:** 10 Targets and annotations.

Target	Annotation
GRM2	Metabotropic glutamate receptor 2; inhibits adenylate cyclase; may regulate neurotransmission and synaptic stabilization
GRM5	Metabotropic glutamate receptor 5; activates calcium signaling; important for synaptic plasticity and neural network modulation
GRM3	Metabotropic glutamate receptor 3; inhibits adenylate cyclase
GRM8	Metabotropic glutamate receptor 8; inhibits adenylate cyclase through G protein signaling
GRM1	Metabotropic glutamate receptor 1; activates calcium signaling; involved in hippocampal potentiation and cerebellar depression
GRM4	Metabotropic glutamate receptor 4; inhibits adenylate cyclase
GRIA2	Glutamate receptor 2; ligand‐gated ion channel for excitatory synaptic transmission in the CNS
SLC1A2	Excitatory amino acid transporter 2; sodium‐dependent transporter for L‐glutamate and aspartate
GRIK5	Ionotropic glutamate receptor kainate 5; mediates excitatory neurotransmission
GRIN1	NMDA receptor 1; calcium‐permeable ion channel with magnesium‐dependent gating

The PPI network diagram emphasizes high scores for several glutamate metabotropic receptors (GRMs) such as GRM2, GRM5, GRM3, GRM8, GRM1, and GRM4, alongside other relevant proteins such as GRIA2, SLC1A2, GRIK5, and GRIN1. These receptors are known to play diverse roles in neuronal cells, yet their specific contributions to processes related to PC have received comparatively less attention. In particular, experience has shown that mGRMs is a major mediator of glutaminergic signaling in many cancers.^110^, ^111^, ^112^ The GRM protein family comprises eight members, from GRM1 to GRM8, all belonging to the G‐protein‐coupled receptor (GPCR) class. These proteins are categorized into three subgroups based on similarities in amino acid sequences and pharmacological properties. Group I includes GRM1 and GRM5, which couple with Gq/11 proteins. Group II consists of GRM2 and GRM3, which couple with Gi/o proteins to inhibit cyclic adenosine monophosphate (cAMP) formation. Group III encompasses GRM4, GRM6, GRM7, and GRM8, which couple with both Gi and Go proteins.^113^ High‐throughput genomic studies have identified susceptibility genes for GRM1, GRM3, GRM4, and GRM8 in non‐small cell lung cancer (NSCLC), melanoma, osteosarcoma, and bladder cancer.^114^, ^115^, ^116^, ^117^ The coupling of GRM proteins with GPCRs facilitates the transmission of signals to secondary messengers and downstream pathways, leading to slower physiological responses. Six of the target genes enriched were in the GRM family, indicating that DON is closely related to glutamate metabolic pathway in PC, and there are potential therapeutic targets in this pathway. GRIA2, through its role in regulating calcium influx and downstream signaling pathways, could potentially affect cellular processes relevant to cancer biology, such as proliferation, apoptosis, and migration. Although specific studies directly linking GRIA2 to PC are limited, exploring its expression patterns and functional relevance in cancer cells could provide valuable insights into its potential as a biomarker or therapeutic target. Studies have additionally indicated that GRIA2 signaling plays a role in regulating the expression of genes associated with the MAPK/ERK signaling pathway. This pathway is crucial for cellular processes such as cell proliferation, differentiation, and survival.^118^ The involvement of GRIA2 in modulating this pathway suggests a potential mechanism through which it could influence cellular behavior beyond its traditional neurological functions. In conclusion, although these target genes to PC biology are still being elucidated, their roles in other cancers and neuronal systems provide a foundation for further exploration.

##### 4.1.2.3 GO analysis and KEGG pathway analysis

**FIGURE 4:**
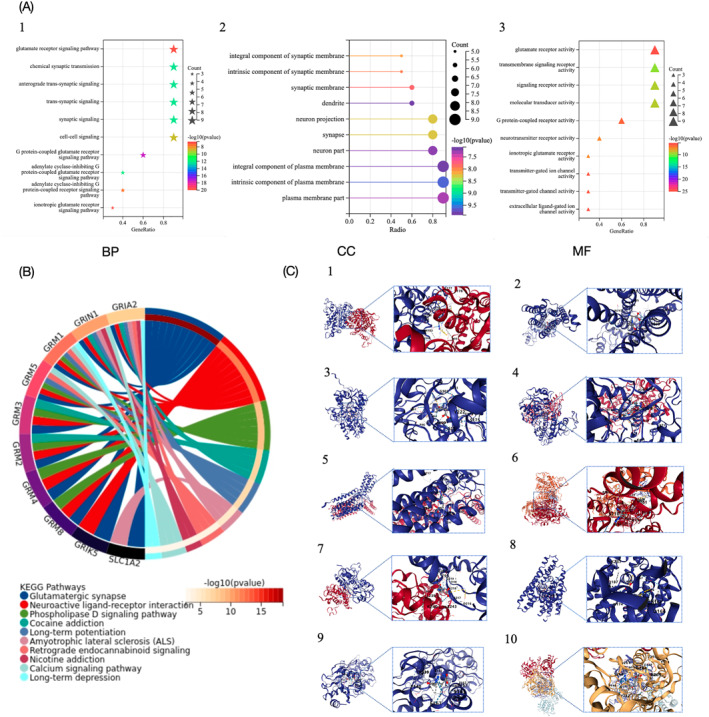
Bioinformatics analysis based on target genes of DON. (A) GO enrichment analysis of core targets based on DON. (B) Enrichment analysis of KEGG pathway based on DON core targets. (C) Molecular docking diagram of DON with core targets. 1. GRM2; 2. GRM5; 3. GRM3; 4. GRM8; 5. GRM1; 6. GRM4; 7. GRIA2; 8. SLC1A2; 9. GRIK5; 10. GRIN1.

GO analysis and KEGG pathway analysis were conducted on the 10 targets gene (Table 1). A threshold of *p* < 0.05 was set, with the top‐ranking enrichment results selected and divided into three categories: biological process (BP), molecular function, and cellular component (CC). These results are exhibited in Figure 4A.

BP categories provide the best representation of changes in biological functions within the body. To understand the possible mechanisms of DON for PC, a threshold of *p* < 0.05 was set. The top 10 key signaling pathways were highlighted using loop graph (Figure 4B).

The main enrichment pathways are glutamate receptor signaling pathway. Research into the role of glutamate receptor signaling pathways in PC is currently advancing. Glutamate receptors, including NMDA (N‐methyl‐D‐aspartic acid) receptors and metabotropic glutamate receptors, are being investigated for their expression in PC cells and their potential involvement in tumor progression.^119^ Activation of these receptors may influence cellular behaviors such as proliferation, migration, and invasion, impacting the aggressive nature of pancreatic tumors. Moreover, these receptors could modulate the tumor microenvironment, affecting interactions with stromal and immune cells. Targeting glutamate receptors or their downstream pathways presents a promising avenue for therapeutic intervention in PC, potentially enhancing treatment strategies and patient outcomes. However, further research is needed to elucidate the exact mechanisms and validate these findings for clinical application.

##### 4.1.2.4 Molecular docking

According to the results of the PPI and KEGG analyses, the compound structural formulas were sourced from the GeneCards website (https://www.genecards.org/) and saved in mol2 format. 3D structure maps of receptor proteins were acquired from the PDB database and selected based on X‐ray crystallography data and Angstrom (Å) value, then saved in PDB format. Cavity‐detection guided Blind Docking analysis was performed using the online tool CB‐DOCK2 (https://cadd.labshare.cn/cb‐dock2/php/index.php) to elucidate the interactions between DON and core targets. Binding energy, cavity volume, and center coordinates (*x*, *y*, *and z*) data were then collected to assess binding capacity. The molecules docked with energy less than −5.0 kJ/mol suggest a good binding ability. The results of the molecular docking analyses are presented in Table 2 and Figure 4C.

**TABLE 2:** Molecular docking results between DON and core targets.

Protein	PDB ID	Binding energy (kcal/mol)	Cavity volume (Å3)	Center (*x*, *y*, *z*)
GRIA2	8I0B	−5.3	6879	15, ‐17,‐19
GRIK5	3OM0	−5.2	217	4,51,‐20
GRIN1	5EWJ	−6.1	5462	35, 33, 35
GRM1	4OR2	−6.0	1714	14, 18, 29
GRM2	4XAQ	−5.3	10, 533	6, 43, 24
GRM3	4XAR	−6.6	1229	55,‐31,11
GRM5	4OO9	−5.7	802	−21,‐6,49
GRM4	7E9H	−5.7	11, 105	129,228,163
GRM8	6E5V	−5.4	2893	36,‐74,‐9
SLC1A2	7VR7	−5.6	359	135,160,157

The findings revealed that the binding energies of these targets genes were all lower than −5.0 kJ/mol, indicating a good binding ability. It is pretty clear that GRIN1 binds most strongly with DON, with a binding energy of −6.1 kJ/mol. GRIN1 is a gene that codes for a part of the NMDA receptor, specifically the Glutamate Ionotropic Receptor NMDA Type Subunit 1, which plays a key role in nerve cell signaling.^120^ NMDA receptors are special channels in nerve cells that play a big role in how the brain changes and adapts, which is important for learning and memory. The GRIN1 gene helps make sure these receptors work properly. When glutamate, a chemical messenger, activates these receptors, they open up and allow calcium and sodium ions to flow into the cell, helping neurons send signals.^121^ Right now, there is not much research showing how GRIN1 might be linked to cancer.

### 4.2 How the targeted solution of Gln antagonists in vivo addresses the problem of drug resistance

Innovative delivery systems have the potential to improve the precision and effectiveness of Gln antagonists while minimizing associated toxicity. These systems may involve nanomaterials, nucleic acids, ferritin carriers, exosomal vehicles, and other advanced methods. By using these advanced delivery approaches, medications can be targeted more accurately to specific tissues or cells, enhancing therapeutic outcomes. This helps to minimize side effects on healthy parts of the body. Plus, these systems can make drugs stay in the bloodstream longer and work better overall by increasing how much of the drug gets into the body and how stable it is. Innovative delivery platforms, such as biomimetic carriers and programmable nanostructures, further enhance these capabilities.^122^ Drug delivery technology can enhance drug target delivery, minimize drug off‐target effects and improve patient compliance.

#### 4.2.1 Nanomaterial‐based delivery systems

Nanomaterials have been extensively explored in the design of drug nanocarriers. More than 50 nanoformulations, including liposomes, polymers, and albumin nanoparticles (NPs), have been approved for clinical use, mainly for cancer treatment.^123^, ^124^ For instance, galactosylated nanoparticles responsive to reactive oxygen species (ROS) have shown significant promise in delivering doxorubicin to TNBC cells, leveraging the high oxidative stress within tumor environments for targeted drug release, as demonstrated by recent studies.^125^ Similarly, ROS/glutathione‐sensitive nanoparticles with miR155 and curcumin enhance immune modulation and chemotherapy to overcome drug resistance, representing an innovative approach in leveraging oxidative stress and redox balance for targeted drug delivery.^126^ Using nanoparticles as carriers, targeted delivery to DON can be achieved by regulating the particle size, surface properties, and composition. For example, researchers have optimized nanoparticle systems with pH‐sensitive release mechanisms that activate preferentially in acidic tumor microenvironments, enhancing localized delivery.^127^, ^128^ Additionally, magnetic nanoparticles have demonstrated excellent potential for externally controlled drug release, providing a promising direction for future exploration.^129^ It is noteworthy that PEGylated liposomes co‐encapsulating gemcitabine and DON achieve prolonged circulation, enhanced tumor accumulation, and synergistic inhibition of PDAC xenograft growth, underscoring the clinical translatability of this combination approach.^130^, ^131^ Studies have shown that when porous Pt‐Pd nanoflowers (Pt‐Pd NFs) are combined with DON (Pt‐Pd@DON), they promote dendritic cell maturation and CD8 T cell infiltration under endogenous H_2_O_2_, thereby alleviating hypoxia and accurately delivering drugs to diseased tissues or cells.^132^ This approach improves therapeutic efficacy and reduces adverse effects on healthy tissue, demonstrating potential for clinical development, especially in treating large PC tumors.

#### 4.2.2 Nucleic acid‐based delivery systems

There are several nucleic acid drugs, including antisense oligonucleotides (ASO), small interfering RNA (siRNA), microRNA (miRNA), aptamers, bait (decoys), and CpG oligodeoxynucleotide.^133^, ^134^, ^135^ Recent advances in CRISPR/Cas systems have further expanded the toolbox for nucleic acid‐mediated therapies, offering highly precise genetic interventions.^136^ By designing specific nucleic acid sequences to deliver DON antagonists, these systems can efficiently target and regulate gene expression in cancer cells, enabling robust therapeutic outcomes.

#### 4.2.3 Ferritin‐based delivery systems

Ferritin can be used as nanocarriers to package multiple drugs for tumor therapy. Common drugs include cisplatin, Gd‐DO3A, and deferriamine B.^137^ Moreover, ferritin can be targeted by peptides or ligand molecules that bind to tumor‐specific receptors. Other molecules such as polyethylene glycol, fluorescent dyes and antibodies can also be attached to the ferritin surface. In addition, ferritin can be loaded with imaging agents, iron oxide, and nucleic acids (such as siRNA).^138^, ^139^ Therefore, ferritin can encapsulates multiple drugs for tumor delivery in nanotherapy, but ferritin is not yet used to deliver Gln antagonists, and its nanotherapy and wrapping in ferritin may require more research and development. Future research should focus on optimizing ferritin's biocompatibility and enhancing its binding affinity to Gln antagonists through advanced molecular docking simulations and structural engineering techniques.^140^ This may involve determining the chemical properties of Gln antagonists suitable for ferritin binding and achieving stable drug‐loading systems at the nanoscale. Nonetheless, with continuous advances in nanotechnology and increasing demand for tumor therapy, research into ferritin as a potential drug delivery vehicle is ongoing, and more studies targeting Gln antagonists may emerge in the future.

#### 4.2.4 Exosome‐based delivery systems

Exosomal delivery systems represent a promising avenue for the precise delivery of Gln antagonists in challenging cancer environments, such as PC.^141^ Exosomal delivery of Gln antagonists may have potential therapeutic effects in PC treatment. Exosomes can act as delivery vehicles to transport anti‐cancer drugs, such as chemotherapy agents. Besides, specially designed nanoformulations that respond to ROS have been shown to be effective by taking advantage of the unique features of the tumor environment.^142^ In PC, the tumor microenvironment (TME) is characterized by a dense network of connective tissue growth and an abnormally high amount of extracellular matrix (ECM).^143^ Using exosomes to deliver glutamine (Gln) antagonists might help by interfering with the tumor cells' metabolism, potentially offering a new therapeutic approach. On top of that, modifying the surface of exosomes with specific targeting molecules such as ligands or peptides could help them deliver their cargo more efficiently and precisely.^144^ Exosomes can also act as transporters, delivering not just their natural contents but also other anti‐cancer agents, such as chemotherapy drugs, to boost treatment effectiveness. For instance, combining exosomes with advanced nanotechnologies—such as polymer‐based particles or magnetic nanoparticles—could be a really promising way to deliver multiple drugs at once.^145^, ^146^ Meanwhile, exosomes seem capable of adapting to the tumor microenvironment, especially in PC, which means they might be able to overcome various physical and chemical barriers there. This adaptability really emphasizes their potential as versatile delivery tools that can navigate complex tumor environments.^147^

#### 4.2.5 Challenges and future perspectives

These delivery systems hold promising potential, but there are still some challenges to overcome when it comes to making them stable, scalable, and affordable enough for real‐world clinical use. Moving forward, it is important to explore ways to combine these systems with other therapies. For example, pairing glutamine (Gln) antagonists with immune checkpoint inhibitors might lead to better treatment results. We also need more research to understand how tumor cells and the tumor environment resist these Gln‐targeting approaches, so we can develop more effective strategies.

### 4.3 Clinical progress, efficacy, and challenges in Gln‐targeted agents

In the Phase II trial NCT03965845, adding CB‐839 (telaglenastat) to nab‐paclitaxel showed some synergistic activity but was limited by gastrointestinal toxicity and only small gains in progression‐free survival.^148^ DRP‐104, now in a Phase I/II study (NCT04471415), has been well tolerated so far with early signs of antitumor effect.^149^ In PDAC mouse models, the Gln antagonist prodrug JHU‐083 enhanced anti‐PD‐1 therapy by cutting down suppressive myeloid cells and boosting CD8^+^ T‐cell infiltration, supporting a combined Gln ‐inhibitor plus checkpoint‐blockade approach. These trials show that while Gln metabolism can be targeted in patients, clinical benefit has been modest and treatment‐related toxicity remains a barrier to wider application. As shown in Table 3, these strategies balance unique advantages—improved tumor penetration, immune regulation and precise metabolic blocking ‐ with challenges such as adaptive resistance, off‐target toxicity, and matrix barrier. These insights form a coherent framework for developing collaborative combination schemes and precise delivery systems in future clinical applications.

**TABLE 3:** Strategies for the treatment and management of pancreatic cancer.

Strategy	Details	Advantages	Challenges
Surgical resection	Radical surgical resection remains the most effective treatment for PC	Provides potential for curative outcomes in resectable cases	High recurrence rate; limited to early‐stage patients; poor overall survival rate post‐surgery
Chemotherapy regimens	Includes gemcitabine, FOLFIRINOX, and the newer NALIRIFOX	Proven to increase survival; combinations such as nab‐paclitaxel/gemcitabine enhance efficacy	Drug resistance due to EMT and TME adaptations
Targeting Gln metabolism	Use of Gln antagonists such as DON and its prodrugs (e.g., JHU‐083, DRP‐104) to inhibit Gln‐dependent metabolic pathways in tumor cells	Reduces tumor growth and reshapes immune cells; enhances chemotherapy sensitivity	Drug resistance; potential systemic toxicity; limited data in PC‐specific applications
Innovative drug delivery systems	Incorporates nanomaterials, nucleic acids, ferritin, and exosomal vehicles to enhance targeted delivery of therapies like DON	Improves bioavailability and circulation time; reduces off‐target effects; enables targeted delivery to TME	Optimization of stability, scalability, and cost; limited clinical data
Antibody drugs targeting Gln	Includes tumor autoantibodies targeting specific pathways in Gln metabolism	High specificity; potential for combination therapy; reduces off‐target effects	Immune tolerance; complexity of antibody‐drug development; potential resistance mechanisms
Targeting the TME	Strategies to disrupt the fibrotic barrier, reduce ECM production, and counteract hypoxia‐induced drug resistance. Includes CAF‐targeting therapies and immune checkpoint inhibitors	Enhances chemotherapy efficacy; remodels the TME to improve drug penetration and immune cell infiltration	Complex interactions in the TME; balancing effective targeting without exacerbating immunosuppression
Combination therapies	Combines Gln metabolism inhibitors with chemotherapy, radiotherapy, or immunotherapy	Enhances overall efficacy by targeting multiple pathways; potential for reducing drug resistance	Risk of additive toxicities; requires personalized approaches
CRISPR/Cas‐based nucleic acid delivery	Utilizes advanced genetic editing tools for highly precise interventions in Gln metabolic pathways	Precise targeting; enables genetic modification of resistant tumor cells	Ethical and regulatory concerns; scalability for clinical use

## 5 DRUG DEVELOPMENT POTENTIAL FOR ANTIBODIES AGAINST Gln

### 5.1 Advantages and application prospects of antibody drugs

Antibody drugs have many advantages and broad application prospects. They are highly specific and selective and can achieve precise therapeutic effects by binding with specific antigens, and reducing the effects on normal cells. Antibody drugs often have lower toxic side effects than traditional chemical drugs because they act more specifically on specific molecules or cells. Moreover, antibody drugs are long‐acting and regulatory, which can persist in vivo for a period of time and be regulated by adjusting the dose or structure. Antibody drugs work in many areas. They help treat tumors, autoimmune disorders, infections, and neurological conditions. In PC, antibodies can target glutamine metabolism. They block key enzymes or transporters. This attack starves cancer cells and slows their growth. Their precision may reduce side effects. Combining these antibodies with other treatments can improve results.

Challenges still exist. Cancer cells may develop resistance. Patients can respond differently. Safety concerns also need careful study. Despite this, antibody‐based glutamine targeting offers a promising new avenue for PC therapy.

### 5.2 Role and progress of tumor autoantibodies in the treatment of PC

PC is aggressive and often deadly. Current treatments have limited success. Recently, researchers have turned to tumor‐associated autoantibodies (TAAbs). These are immune proteins that target markers on PC cells. TAAbs may help the body fight this disease more effectively.^150^, ^151^ Studies have found that TAAbs can fight tumors in a few different ways. TAAbs recognize specific markers on cancer cell surfaces. This binding activates the immune system and directs it to destroy those cells. TAAbs also inhibit tumor growth by blocking cancer cell division and invasion. In addition, they stimulate immune cells and strengthen the overall immune response against tumors.^152^ Previous studies have found that seven TAAbs (TOR1B, RNF138, PPP1R15A, PAICS, LENG1, GPR3, and CYP3A5) can be used to assist the diagnosis of PC, pancreatitis, and immune pancreatitis (AUC = 0.882‐0.940).^153^ Five TAAbs (anti‐CLDN17, anti‐KCNN3, anti‐SLAMF7, anti‐SLC22A11, and anti‐OR51F2) by human protein microarray chip as novel diagnostic biomarkers for PC.^154^ In addition, through protein microarray screening and ELISA method, the research team found 10 important TAAbs and showed good sensitivity and specificity as a candidate biomarker for the diagnosis of PDAC.^155^ Drawing on the methods described in the above studies, if certain TAAbs related to the Gln metabolic pathway are found, attempts can be made to develop therapies against these antibodies. To examine the association between these TAAbs and patient clinical characteristics, response to treatment, and prognosis. This can help to determine whether these antibodies can be used as potential biomarkers for tumor diagnosis, prognostic evaluation, or monitoring of therapeutic response 3.

In PC treatment, some tumor autoantibodies have been demonstrated with potential therapeutic effects. CA19‐9, carcinoembryonic antigen CEA, glycotype antigen 125 are commonly used PC markers among which CA19‐9 is the most valuable tumor marker in PC, and the production of autoantibodies is related to the development and prognosis of PC.^156^, ^157^, ^158^ However, the study of TAAbs in the treatment of PC is still in the early stage, and further studies and clinical trials are still needed to verify their efficacy and safety. In addition, the application of TAAbs also faces some challenges, such as antibody selection, immune tolerance and other issues. Overall, tumor autoantibodies have a potential role in PC treatment, but further studies are needed to further understand the mechanisms and to develop more effective therapeutic strategies.

### 5.3 Current status and challenges of antibody drugs against Gln

Currently, most studies have focused on the role of Gln in cancer cells and how to effectively target this pathway. Scientists are working to identify and validate key enzymes and proteins in Gln metabolism as potential drug targets. A few studies have entered the preclinical phase, testing the impact of specific antibody drugs on cancer models. For example, an ASCT2‐targeting ADC, MEDI7247, demonstrated manageable thrombocytopenia and neutropenia along with a 16.4% objective response rate in a first‐in‐human Phase 1 trial of relapsed/refractory hematologic malignancies. These findings support the development of similar ASCT2‐ADC strategies to exploit Gln dependency in solid tumors such as PDAC, pending dedicated preclinical evaluation.^159^, ^160^ Additional SLC1A5‐directed ADCs and blocking antibodies are in preclinical development, showing potent inhibition of Gln uptake and tumor growth in PDAC models.

However, there are still many unsolved problems, such as Gln metabolism involving multiple pathways and enzymes, which increases the complexity of drug development and requires precise target selection and regulation strategies. Cancer cells may develop resistance to antibody drugs, which calls for ongoing research and the development of new drugs. These models help scientists see how well drugs work. Researchers must still evaluate their effects on normal cells. This step ensures treatment safety. Preclinical models play a key role before clinical trials. These models let scientists test drug efficacy. They also reveal potential side effects. These data guide decisions about moving forward.

## 6 CONCLUSION

Gln metabolism in PC is complex. It plays several roles in the tumor microenvironment. Glutamine fuels cancer cells with energy. It also provides building blocks for DNA, lipids, and proteins. These functions support tumor growth and survival. Glutamine helps maintain redox balance. It influences key signaling pathways. It even affects immune responses. Targeting glutamine use in cancer cells could yield new treatments. Disrupting their energy supply and biosynthesis may slow tumor growth.

Glutamine inhibitors have shown promise in cancer therapy. Some agents are already in clinical trials. Researchers are developing small molecules that block glutamine‐metabolizing enzymes. They are also exploring biological methods to disrupt tumor metabolism. Combining glutamine inhibitors with chemotherapy, radiation, or immunotherapy may boost effectiveness. Personalized medicine really makes a big difference. Profiling each tumor's glutamine metabolism can guide therapy. Patient genetics should also inform treatment choices.

Future studies must confirm safety and find optimal doses. Collaboration among researchers, clinicians, policymakers, and patients is essential. Scaling up production of successful agents will be key. Biomarkers of glutamine dependency could improve early diagnosis. They could also track how well treatments work. Understanding glutamine metabolism may improve outcomes for PC patients. It could also inform strategies against other cancers. This knowledge paves the way for more targeted personalized therapies.

## AUTHOR CONTRIBUTIONS

**Min Su**: Data curation; visualization; writing—original draft. **Huan Qin**: Methodology; visualization. **Jie Shen**: Methodology; visualization. **Hao An**: Methodology; visualization. **Yu Cao**: Conceptualization; writing—review and editing.

## CONFLICT OF INTEREST STATEMENT

The authors declare no conflicts of interest.

## ETHICS STATEMENT

This manuscript is a literature review and does not report on original research involving human participants, human tissue, or animals; accordingly, no ethics approval or consent to participate was required.

## INFORMED CONSENT STATEMENT

Not applicable.

## Data Availability

Data sharing is not applicable to this article as no new data were created or analyzed in this study.
